# Supplementary material for: Causal Relationship between Aging and Anorexia Nervosa: A White-Matter-Microstructure-Mediated Mendelian Randomization Analysis
Source: Biomedicines. 2024 Aug 16;12(8):1874. doi: 10.3390/biomedicines12081874 (PMC11351342; doi:10.3390/biomedicines12081874)
Supplement: Supplementary file 1 [file biomedicines-12-01874-s001.zip › Supplementary Figure/Figure S8/Leaveoneout plot of MR analysis for TL to ubm-b-1568.pdf]

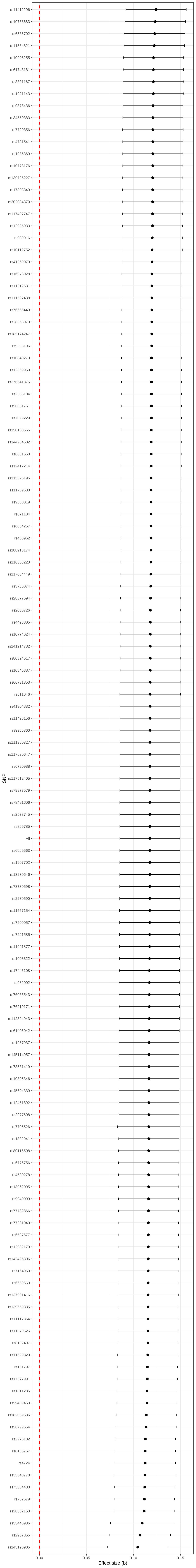
rs11412296
rs10768683
rs6536702
rs11584821
rs10905255
rs61748181
rs3891167
rs1291143
rs9878436
rs34550383
rs7790856
rs4731541
rs1985369
rs10773176
rs139795227
rs17803849
rs202034370
rs117407747
rs12925933
rs939916
rs10112752
rs41269079
rs16978028
rs11212631
rs111527438
rs76666449
rs28363070
rs185174247
rs9398196
rs10840270
rs12369950
rs376641875
rs2555104
rs56061761
rs7099229
rs150150565
rs144204502
rs6881568
rs12412214
rs113525195
rs11769630
rs9600019
rs871134
rs6054257
rs450962
rs188918174
rs116863223
rs117034449
rs3785074
rs28577594
rs2056726
rs4498805
rs10774624
rs141214782
rs80324517
rs10845387
rs66731853
rs611646
rs41304832
rs11426156
rs9955360
rs111950327
rs117630647
rs6790988
rs117512405
rs79977579
rs78491606
rs2538745
rs869785
All
rs6669563
rs1907702
rs13230646
rs73730598
rs2230590
rs11557154
rs7209057
rs7221585
rs11991877
rs1003322
rs17445108
rs932002
rs76065543
rs76219171
rs112394943
rs61405042
rs1957937
rs145114957
rs73581419
rs10805346
rs45604339
rs12451892
rs2977608
rs77705526
rs1332941
rs80116508
rs6776756
rs4530278
rs13062095
rs9940099
rs77732866
rs77231040
rs6587577
rs12932179
rs142426306
rs7164950
rs6659669
rs137901416
rs139669835
rs11117354
rs11579626
rs8102497
rs11699829
rs131797
rs17677991
rs1611236
rs59409453
rs182059586
rs56799554
rs2276182
rs8105767
rs4724
rs35640778
rs75664430
rs762679
rs28502153
rs35446936
rs2967355
rs143190905
SNP
0.00
0.05
0.10
0.15
Effect size (b)
